# A mechanism for punctuating equilibria during mammalian vocal development

**DOI:** 10.1371/journal.pcbi.1010173

**Published:** 2022-06-13

**Authors:** Thiago T. Varella, Yisi S. Zhang, Daniel Y. Takahashi, Asif A. Ghazanfar

**Affiliations:** 1 Department of Psychology, Princeton University, Princeton, New Jersey, United States of America; 2 Princeton Neuroscience Institute, Princeton University, Princeton, New Jersey, United States of America; 3 Department of Ecology & Evolutionary Biology, Princeton University, Princeton, New Jersey, United States of America; Max Planck Institute for Psycholinguistics, NETHERLANDS

## Abstract

Evolution and development are typically characterized as the outcomes of gradual changes, but sometimes (states of equilibrium can be punctuated by sudden change. Here, we studied the early vocal development of three different mammals: common marmoset monkeys, Egyptian fruit bats, and humans. Consistent with the notion of punctuated equilibria, we found that all three species undergo at least one sudden transition in the acoustics of their developing vocalizations. To understand the mechanism, we modeled different developmental landscapes. We found that the transition was best described as a shift in the balance of two vocalization landscapes. We show that the natural dynamics of these two landscapes are consistent with the dynamics of energy expenditure and information transmission. By using them as constraints for each species, we predicted the differences in transition timing from immature to mature vocalizations. Using marmoset monkeys, we were able to manipulate both infant energy expenditure (vocalizing in an environment with lighter air) and information transmission (closed-loop contingent parental vocal playback). These experiments support the importance of energy and information in leading to punctuated equilibrium states of vocal development.

## Author summary

Species can sometimes evolve suddenly; their appearance is preceded and followed by long periods of stability. This process is known as “punctuated equilibrium”. Our data show that for three mammalian species—marmoset monkeys, fruit bats, and humans—early vocal development trajectories can also be characterized as different equilibrium states punctuated by sharp transitions; transitions indicate the advent of a new vocal behavior. To better understand the putative mechanism behind such transitions, we show that a balance model, in which variables trade-off in their importance over time, captured this change by accurately simulating the shape of the developmental trajectory and predicting the timing of the transition between immature and mature vocal states for all three species. Two variables—energy and information—were hypothesized to trade-off during development. We tested and found support for this hypothesis in analyses of two marmoset monkey experiments, one which manipulated energy metabolic costs and another which manipulated information transmission.

## Introduction

The sudden appearance of a new species preceded and followed by periods of relative stability is known as “punctuated equilibria” (i.e., periods of equilibrium punctuated by abrupt changes) [1, 2]. This theory accounts for the appearance of new species in a manner that is different from the gradual change that we normally associate with evolution [1]. More recently, the theory has been applied to the evolution of communication in humans. In both natural and artificial language evolution, long periods of gradual divergence are interrupted by periods of rapid change [3, 4]. The source of this non-linearity in language evolution is thought to be the balance between optimization and flexibility [4].

Since evolution and development are in many ways similar, contingency-based processes (just on different timescales [5–7]), the punctuated equilibrium framework may also be applicable to behavioral development. By analogy with the rapid formation of a new animal species or languages, contextual changes for a developing individual could suddenly lead to a new behavior (i.e., a new locomotion pattern) while co-existing with other behaviors previously established. In these cases, “context” is any new state the individual may occupy; this could be a new body state (following, for example, the growth of one or more parts of the vocal anatomy) and/ or it could be a new environmental state (for example, changing interactions with caregivers or other members of the social group). Here, we investigate this possibility in the development of vocal behavior and explore putative mechanisms.

We focus on three mammalian species—marmoset monkeys, fruit bats, and humans. In all of them, vocal development is influenced by postnatal experience during infancy. Using densely sampled longitudinal vocal recordings, we first demonstrate that gradual vocal development was punctuated by rapid change on the timescale of days. We ask two basic questions: 1) how can we mathematically describe the different species’ vocal developmental trajectories? And 2) are there commonalities among them? We consider three different models of development—linear, recurrent, and balance. Linear and recurrent models are standards in the behavioral development literature. In the linear model, the trajectory is changing at a constant rate. The recurrent model consists of a trajectory that changes until it reaches a stable state and then there is no further change. It is generated by a factor that changes iteratively depending upon a previous time point until it achieves a stable state [8]. Finally, the balance model is one in which change occurs between two stable states and would best represent punctuated equilibria. The balance model can be generated by the weighted sum of two factors whose weights slowly change during development; it is like the double-well potential model used in statistical physics [9].

We find that all three species’ trajectories are best fit by the balance model, the one consistent with punctuated equilibria. We then show that energy and information—and how their importance varies over time for individuals—are good candidates for a contextual change that leads to the sharp transitions observed in vocal behavior. Finally, we test our predictions using new analyses of previously published data from experiments with marmoset monkeys [10,11].

## Results

**Fig 1:**
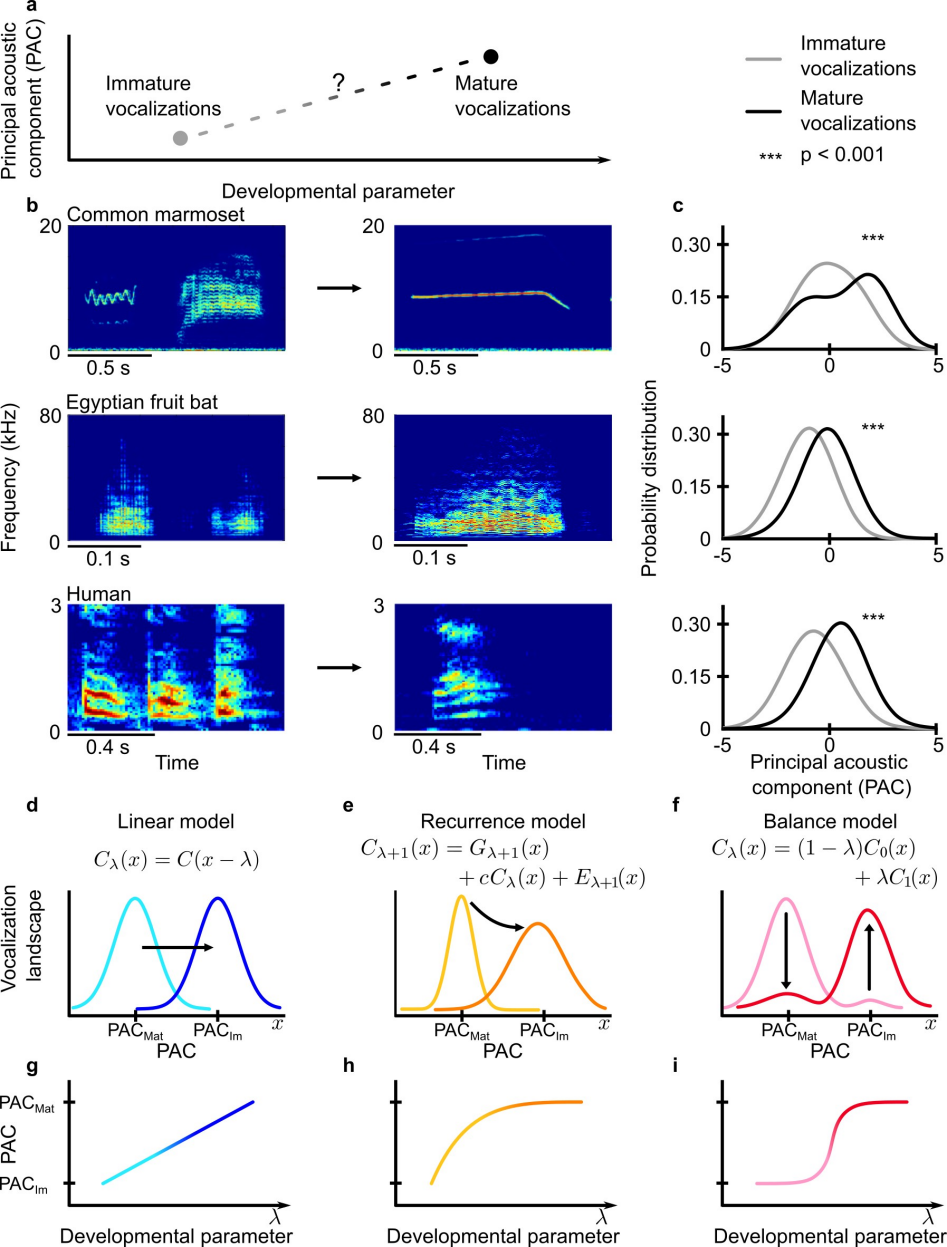
Different models could explain a transition between two developmental states. **a.** Diagram illustrating one hypothetical transition between immature vocalizations to mature vocalizations. The y-axis is the first component of the PCA performed on the vocalizations and labeled as the Principle Acoustic Component (PAC). **b.** Sample spectrograms of vocalization for an infant (immature, left) and an adult (mature, right). From top to bottom, we show an example from common marmoset (*Callithrix jacchus*), Egyptian fruit bats (*Rousettus aegyptiacus*), and humans. **c.** Comparison between the probability distribution of the PACs of immature calls (gray) and mature calls (black). From top to bottom: common marmoset, Egyptian fruit bats, and humans. *** means p < 0.001. **d-f.** Dynamics of the vocalization landscape during the development. The vocalization landscape depends on the probability distribution of the PAC. Lighter colors (light blue, yellow, and pink) represent the immature stage of development, darker colors (dark blue, orange, and red) the mature stage. For an explanation of equations on top of each figure, see Methods - Development models. **g-i.** Transition predicted by each mechanism. The transition is shown by the PAC associated with maximum height in the vocalization landscape throughout development. From left to right: **d,g.** linear model, **e,h.** recurrence model for an asymptotic transition (inspired by [8]) and **f,i.** balance model for a phase transition, based on a non-equilibrium dynamical balance given by the weighted sum of two constraints.

Marmoset monkeys, fruit bats, and humans all use mechanisms for vocal production similar to most mammals [12]. Vocal production results from the interactions among a large number of components: the vocal apparatus (larynx, vocal tract, lungs, etc.), the muscles that move them, the neural circuit activity that leads to muscular contraction, and the organism’s experience that modifies those neural circuits [13]. Monitoring and manipulating all these components are not possible, especially during development. Moreover, at any age, there are an exponentially large number of possible configurations they could take. Measurement of a few key parameters that can account for the shape of a particular vocal developmental trajectory, and that works across species, would be ideal. With this in mind, we analyzed longitudinal datasets freely available to the public [14–17] and measured the changes in the distribution of vocal acoustic features common to our three species’ vocalizations: duration, Wiener entropy, dominant frequency, and dominant frequency of amplitude modulation [17–19]. To reduce the dimensionality of these four vocal parameters, we used principal component analysis to compute their collective first principal component. In our subsequent analyses, we used only this first principal component (“Principal Acoustic Component” or PAC) as it was the component that captured most of the variance of the vocal development dynamics (Fig 1A).

For all three species, the distribution of PACs in earlier versus later periods of development was different (p < 0.001, Kolmogorov-Smirnov test for the equality of probability distribution) (Fig 1B and 1C). We associated a thermodynamic cost function for each probability distribution using the maximum entropy principle [13,20]. The thermodynamic cost is proportional to the negative logarithm of the probability distribution. Then, we defined a two-dimensional landscape as the opposite of the thermodynamic cost. In other words, changes in the probability distribution of vocal acoustics can be interpreted as modifications in the landscape of the vocal production. Thus, we can ask, What kind of changes in the vocalization landscape best describes the trajectory of vocal development? The fit of three different models were tested: linear (Fig 1D and 1G), recurrent (Fig 1E and 1H), and balance (Fig 1F and 1I). The models were chosen based on the behavioral development literature and on the possible behavioral landscape dynamics that could be observed (see Methods - Development models). The linear model is the simplest possible trajectory between two points; it represents a range of psychological and developmental models that (like most evolutionary accounts) involve gradual changes. For example, Piaget [21] and J.J. & E.J. Gibson [22] argued that sensory perception gradually emerges in the human infant. The recurrence model focuses on a curvilinear building-up to a single stable state. The development of mature song in zebra finch via tutoring is captured by this model [8]. Finally, the balance model could account for a nonlinear shift between two stable states; we identified this possibility via its application in the phase-transition literature of physics [9].

**Fig 2:**
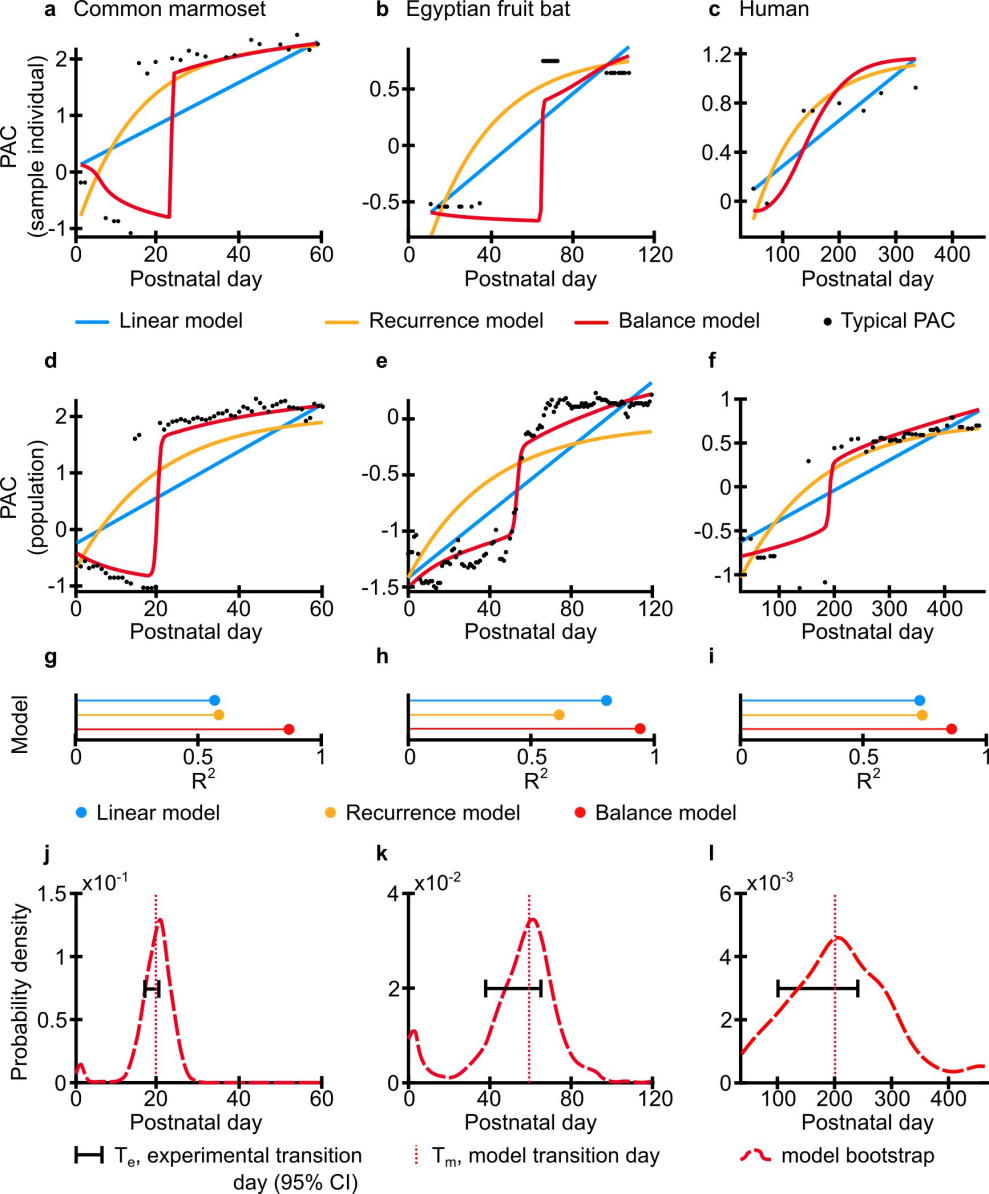
The energy-information balance model most successfully reproduces the transition day between two states for different species. Analysis made using common marmoset in the left column, Egyptian fruit bat in the middle column, and human in the right column. **a-c.** Best model fit for individuals of the three species. Notice that for the bat dataset, the data collection was from different recording periods instead of a single longitudinal experiment; this lead to some gaps in the data. The black dots are the typical PAC, i.e., a moving average calculated from the experimental data per day of recording (see Methods - Estimation of typical PAC per day). Blue lines are the best fit for the linear model, orange lines are the best fit for the recurrence model and red lines are the best fit for the balance model. **d-f.** Model fitting for the population for the three different species. **g-i.** Comparison between the best R^2^ for each model. **j-l.** Comparison of transition date predicted by the balance model and calculated from experimental data. Both transition dates were calculated by fitting a sigmoid to the values, and distributions were obtained via bootstrap.

We fit each of these models to the vocal developmental trajectories of each species to determine which one best captures their shape. Fig 2A–2C shows exemplar trajectories, while Fig 2D–2F show the shape of change across the population (marmosets: n = 10 and 105,904 vocalizations; bats: n = 13 and 1878 vocalizations; humans: n = 8 and 1055 vocalizations) (Fig 2A–2F). For each model, we fit the initial (immature) and the final (mature) landscapes through 3 parameters: the last immature day, the first mature day, and the thermodynamic “temperature” *β* that is important for the relationship between the landscape and the probability distribution (see Methods - Estimation of the vocalization thermodynamic cost and landscape). In this manner, the success of the model would be measured by whether the shape of the trajectory could be predicted using only the extreme data points—the data between the last immature day and the first mature day were not used as inputs. We compared the goodness of fit (adjusted R^2^) of each model to the full data. For all species, the balance model best captured the trajectory of vocal development (adjusted R^2^ respectively for linear, recurrent and balance model: marmosets: 0.54, 0.60, and 0.86; bats: 0.80, 0.63, and 0.96; humans: 0.70, 0.71, and 0.89)(Fig 2G–2I). We performed statistical tests to assess whether the adjusted R^2^ of the balance model was significantly higher than that of the linear and recurrence model for the three species. All were significant (p < 0.05) except for the balance model compared to the linear model in humans (p = 0.093).

If the balance model is accurate, it should also be able to predict when the transition between the two stable states occurs. That transition day is the time when the two landscapes are balanced, i.e., have the same maximum height. To estimate the transition day during vocal development, we fit the experimental data with an S-shaped curve (a sigmoid function). We then tested whether the balance model could correctly estimate the timing of developmental transitions in the vocalization data. For all species, the transition day estimated by the balance model is within the confidence interval of the transition seen in the data (Fig 2J–2L). For marmoset monkeys, the model transition day was 20.07 whereas the experimental transition day was 20.85 (p = 0.528; 95% CI = [17.55, 20.87]). For bats, the model and experimental transition days were 54.22 and 55.88, respectively (p = 0.604, 95% CI = [35.67, 60.55]). For humans, the model and experimental transition days were 191.83 and 173.41 (p = 0.331, 95% CI = [104.30, 238.47]). These data indicate that early vocal development in all three species exhibits punctuated equilibria—equilibrium states separated by a sharp transition.

The balance model provides an account for why there are sudden transitions during vocal development. Knowing how a behavioral landscape changes helps us understand the underlying causes of those transitions. The balance model assumes that the vocalization landscape—a changing context—consists of two components (that are each landscapes as well). Furthermore, it predicts that these two components will trade-off, one increasing and the other decreasing throughout development. What could those components be? One of the most important trade-offs in animal behavior is between metabolic energy and information [23], so that was a logical possibility. In many animals, vocal output is linked to increases in metabolic energy expenditure [24,25]. For example, louder versus softer vocalizations in zebra finches and humans require greater energy expenditures [26,27]. In marmoset monkeys, infant vocal output is tightly correlated with fluctuations of arousal (a marker of energy allocation) [28,29]. With regard to information and vocalizations, there are also many accounts. For example, the crying rate is highest in human infants during the first two months of life during which they have poor control over phonation [30]. As they begin to cry less and produce more steady (tonal) vocal sounds, they more reliably elicit vocal responses from caregivers [31]. The same is true for marmoset monkey infants and the elicitation of responses from caregivers [17,32].

It is important to keep in mind the way we defined the landscape: the peaks in the landscape are associated with the behavior that is produced with higher probability. Moreover, we assume some vocalizations are more energetically costly (as in metabolic cost) than others, and some are more efficient in transmitting information than others. When the energy landscape is at its peak, the assumption in our model is that more energy is being expended on vocalizations as opposed to other behaviors because to do so is less costly. Likewise, when the information landscape is at its peak, we are assuming that the vocalizations are more efficient in transmitting information. If the final landscape is a weighted sum of two landscapes, as described by the balance model, one landscape will start high and decrease, and the other will start low and increase. Thus, for the case of vocal development, we hypothesized that very young animals produce immature vocalizations at *high* rates (higher energetic landscape since vocalizations will be less energetically costly to produce); these vocalizations have *less* information content as they less reliably elicit responses from conspecifics (lower information landscape). As they get older, more mature-sounding vocalizations are produced at *lower* rates (lower energetic landscape) but contain *more* information content with greater likelihood of eliciting a conspecific response (higher information landscape).

**Fig 3:**
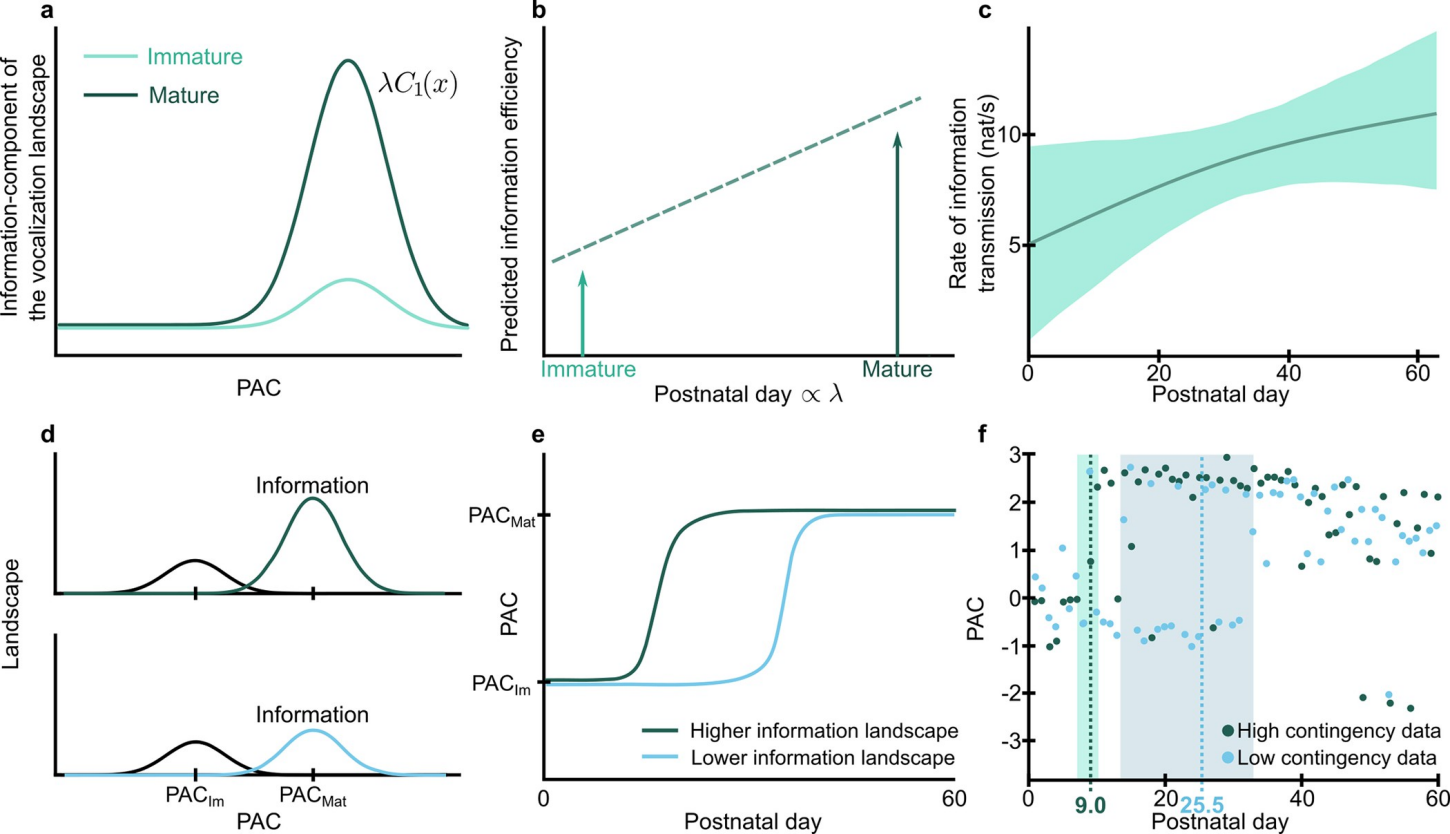
Information transmission efficiency is related to the mature component of the vocalization landscape. **a.** Schematic of how the predicted information component of the vocalization landscape, *λC*_1_(*x*), varies from immature (smaller *λ*) to mature phases (larger *λ*) of development, being lower during the mature stage. *λ* is the parameter that controls the balance between landscapes. *C*_1_(*x*) is the component of the landscape which relevance increases during development. **b.** Expected increase of efficiency in information transmission given the increase in information landscape. **c.** Observed information transmission of marmoset infant calls throughout development. The shaded region represents a 95% confidence interval. **d.** Schematic of two situations with different information component of the landscape *C*_1_(*x*): the plot on the top represents a higher information landscape, the plot on the bottom represents a lower information landscape. The black line represents the energy component, *C*_0_(*x*), and is assumed to be constant in the two scenarios. **e.** Predicted vocal dynamics, measured by the optimal PAC, for the two scenarios, showing that higher information landscape predicts faster transition. **f.** Observed vocal dynamics from the feedback contingency manipulation setup in marmosets. Dashed lines represent transition day T_high_ for high contingency data (dark green line) and T_low_ for low contingency data (light blue line). T_high_ < T_low_ with p < 0.001.

Thus, according to our hypothesis, the information component of the vocalization landscape, *C*_1_(*x*), is initially low (near 0) and then increases due to the changing weight *λ*. A higher information component of the landscape leads to an increase in the information transmission efficiency (Fig 3A and 3B). We tested our hypothesis directly in developing marmoset monkeys by measuring information transmission efficiency via the change in probability of parental responses following an infant vocalization. Using Granger causality, we found that, as marmoset infants get older, their vocalizations elicit parental responses with greater reliability (i.e., information component of the landscape increases and the information transmission efficiency increases; Fig 3C). If information plays a causal role in shaping the vocal developmental trajectory in the manner predicted by the balance model, then changing the transmission of information should alter the timing of the transition between equilibrium states. That is, the punctuation described by the transition day should shift. For example, in a situation where one individual produces vocalizations with higher information transmission efficiency (i.e., the landscape of the information component has a higher maximum (Fig 3D)), the transition from immature to mature vocalization should occur earlier (Fig 3E). We used data from a published experiment that manipulated the degree of parental contingency of vocal feedback that an infant marmoset receives during development to test this “information” hypothesis [10].

In the experiment, there were three pairs of dizygotic twins (6 infants from 3 different sets of parents). Starting at postnatal day 1 (P1), one randomly selected twin was provided the best possible simulated “parent” who gave 100% vocal feedback via a computer-controlled closed-loop playback system when the infant produced an immature contact call. The other twin received vocal feedback to only 10% of the contact calls it produced [10]. This contingency experiment was performed approximately every other day for less than 1 hour after which the infants were returned to their families. In the context of the current study, a higher level of simulated parental responsivity to an infant’s vocalizations is effectively increasing the information landscape and thus should shift the transition day in a manner predicted by the balance model: an earlier transition. The opposite should be true for infants whose vocalizations elicited simulated parent calls with a low probability. As predicted, we observed that the low contingency marmosets do have a significantly later (p < 0.001) transition day than the high contingency marmosets, both estimated by fitting a sigmoid (high contingency data transition day = 9.0, 95% CI = [7.1, 10.2]; low contingency transition day = 25.5, 95% CI = [13.5, 33.7]) (Fig 3F). Statistical tests were performed to check whether the balance model would predict the transition day similarly to what we observed in Fig 2J–2L. The statistical test performed after bootstrapping the transition day given by the balance model and the sigmoid fit showed that they were not significantly different for either the high contingency data (p = 0.284) or low contingency data (p = 0.168). Therefore, using the balance model and different information landscapes, we could predict the qualitative changes in the transition day without any fitting to the data.

**Fig 4:**
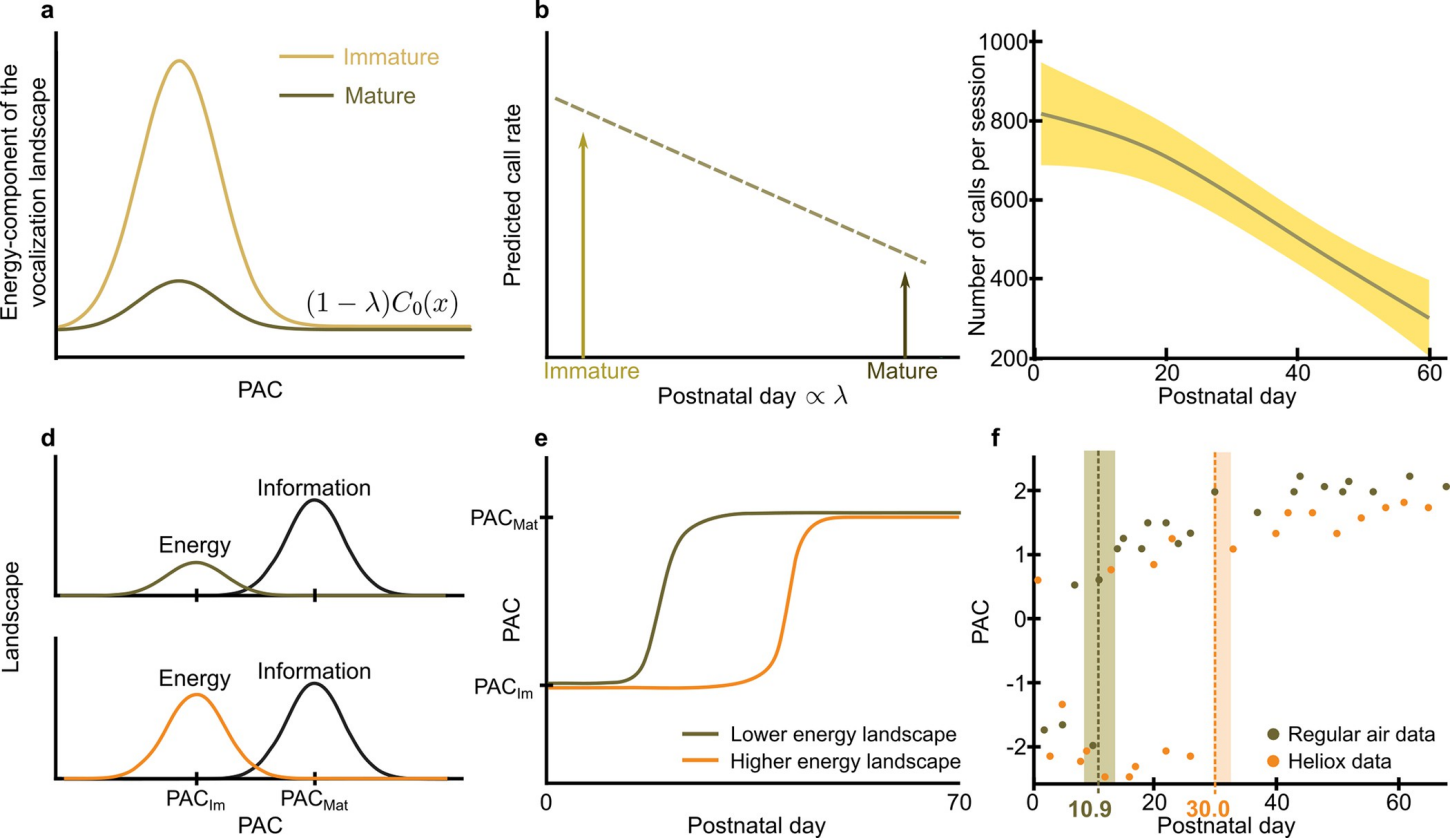
Energy metabolic cost is related to the immature component of the vocalization landscape. **a.** Schematic of how the predicted energy component of the vocalization landscape, (1−*λ*)*C*_0_(*x*), varies from immature (*λ* closer to 0) to mature (*λ* closer to 1) phases of development, being higher during the mature stage. *λ* is the parameter that controls the balance between landscapes. *C*_0_(*x*) is the component of the landscape which relevance decreases during development. **b.** Expected decrease in call rate. **c.** Observed decrease in call rate. The shaded region represents a 95% confidence interval. **d.** Schematic of two situations with different energy component of the landscape *C*_0_(*x*): the plot on the top represents a lower energy landscape; the plot on the bottom represents a higher energy landscape. The black line represents the information landscape, *C*_1_(*x*), and is assumed to be constant in the two scenarios. **e.** Predicted vocal dynamics, measured by the optimal PAC, for the two scenarios, showing that lower energy landscapes predict faster transition. **f.** Observed vocal dynamics from the heliox setup in marmosets. Dashed lines represent transition day T_air_ for regular air data and T_heliox_ for heliox data. T_air_ < T_heliox_ with p < 0.001.

Likewise, according to our hypothesis, the energetic component of the landscape, C_0 (x), is initially high then decreases as the weight (1-λ) changes (Fig 4A), which could be a result in changes of the energetic costs of producing vocalizations. One consequence of that change could be a decrease in call rate (Fig 4B), given that the more the marmoset vocalizes, the more energy it spends. We tested the hypothesis indirectly by measuring the vocalization rate over time. We found that, as the marmoset gets older, the number of vocalizations decreases (Fig 4C). Similar to the manipulation of information landscapes, the manipulation of energy landscapes should also affect the timing of the transition day. If we increase the energy landscape, then it should take longer for both landscapes to balance out: The transition day is predicted to be later (Fig 4D and 4E). For both infants and adult marmoset monkeys, vocal production is dependent upon respiration [17,29,33], as it is for all terrestrial mammals [12,34]. We can manipulate the energy landscape by reducing the effort it takes to respire by placing individuals in a helium-oxygen (heliox) environment. (Indeed, for this reason, it is used by clinicians to treat children with respiratory ailments [35].) The lighter air reduces the energy expenditure for respiration and, logically, for vocalizations as well.

We again used data collected from a published study wherein, for 10 minutes per recording session (every other day for 2 months), infant marmosets were placed in an 80% helium and 20% oxygen environment; the mix is lighter than regular air but has the same concentration of oxygen [11]. Thus, in the brief period in which the air is lighter, the vocalization metabolic cost is reduced. A lowered metabolic cost of producing vocalizations translates to greater frequency of vocal output and an increase in its representation in the landscape. A lower metabolic cost will increase its representation in the landscape (see Methods –Estimation of the vocalization thermodynamic cost and landscape). As such, we predicted that the transition day would be later for those vocalizations of infant marmosets recorded while in the heliox condition versus those recorded while in regular air. [To be clear, we are comparing the vocal developmental trajectory as measured in heliox versus measured in regular air. We are not assessing the long-term influence of heliox on vocal production in regular air.]

Indeed, this is what we observed: the transition day is significantly later for heliox compared to air (p < 0.001; heliox transition day = 30.0, 95% CI = [29.8, 32.6]; regular air transition day = 10.9, 95% CI = [8.5, 13.6]) (Fig 4F). Likewise, the statistical test performed after bootstrapping the transition day given by the balance model and the sigmoid fit revealed that they were not significantly different for vocalizations produced in either regular air (p = 0.61) or heliox (p = 0.092).

## Discussion

Just as evolution occurs when context changes for a population or sub-population of a species, individual behaviors can also change as contexts change. In both cases, “contexts” can include changes in the environment but, importantly, also changes in the morphology and/or internal state of the organism [5,6,36]. When context changes are combined with threshold mechanisms, the sudden changes—relative to the gradual changes typically associated with evolution and development—are possible. Our data show that for three mammalian species—marmosets, fruit bats, and humans—early vocal development trajectories can be characterized as different equilibrium states punctuated by a sharp transition. The balance model captured this change by accurately simulating the shape of the developmental trajectory and predicting the timing of the transition between immature and mature vocal states for all three species. Energy and information trade-off in all sensorimotor systems in all species and thus, it made sense to use these variables. Our empirical data from marmoset monkeys supported the notion that the weights of the energy and the information components of the landscape do indeed shift in opposite directions during vocal development: the energetic component increases over developmental time while the information component decreases. This had the effect of reducing the rate of vocalizations while the effectiveness of infant vocalizations to elicit parental responses increased. To show a causal link between the energy-information trade-off, we manipulated each, revealing that the transition timing between equilibrium states shifted in the direction predicted by the balance model.

There are, of course, other instances of sharp transitions during behavioral development. Another type of motor development—prehension—exhibits similar, self-organized rapid transitions. Human infants make a switch from non-reaching to reaching early in postnatal life [37]: From about 8 to 14 weeks, human infants will reach for an object without grasping, then over the course of only a week or so, they switch to reaching with grasping [38]. Is labeling these development trajectories with abrupt transitions using the evolutionary term “punctuated equilibrium” valid? In evolution, punctuated equilibrium refers to the sudden appearance of a new species preceded and followed by periods of relative stability [1]. Gould and Eldridge argued that it accounts for the appearance of new species in a manner that is different from the gradual change that we normally associate with evolution [1]. This theory holds that sometimes small sub-populations of species may enter a new context (or “landscape” à la Waddington [39]), allowing for reproductive isolation. For example, in the evolution of horses, multiple species rapidly branched from a single lineage with these species co-existing with each other for some time [40]. In the case of ancestral humans, this might account for the co-existence of Neanderthals with linguistically-capable, anatomically-modern humans for some period of time [41] (but see [42] for the possibility of Neanderthals with language capabilities). In our view, vocal development in an individual follows a similar trajectory. There is an initial set of vocal behaviors produced by infants and suddenly there is a new mode of behavior that doesn’t necessarily replace (or is a transformation of) the earlier vocal behaviors. For example, infant marmosets produce a number of vocalizations early in life but their contact calling is infrequent, unreliable and noisy. However, later, they will rapidly transition to produce adult-like contact calls and produce them only in a specific context [13,17,29,43]. These contact calls are now produced along with other calls that were present in the repertoire earlier in life (e.g., twitter calls, alarm calls, etc.). Thus, in this sense, contact calls (a “species” of vocalizations) now coexists with others that were there before. Such an interpretation is consistent with the idea that evolution and development are both contingency-based, historical processes but operate on different time scales [6].

Our longitudinal data account for the punctuated trajectory of one set of vocalization variables in a particular age range and timescale. Just as there is in evolution, there are other modes of change in development consistent with the other two models we tested. Supporting the linear model (Fig 1C and 1D), a study of syllable-order development in juvenile songbirds (zebra and Bengalese finches) and humans infants showed that syllable order changes only very slowly and gradually [44]. Consistent with the recurrent model (Fig 1E and 1F), zebra finches also exhibit an example of diminishing return changes, but over generations [8]. Individual zebra finches learning a song from a tutor who produced a simple, non-wildtype song nevertheless showed an initial bias (i.e., strong change) towards learning certain wildtype-like syllables. That bias decreased over generations but eventually led the song to sound more and more like the wildtype song [8]. Thus, there was an initial strong change in vocal behavior followed by a slower adaptation to a stable point. While songbirds are an outstanding model for vocal learning, we did not include them in our study for two reasons, one practical and one anatomical. The practical reason is that we were unable to find a data set that tracked the development of their non-song vocalizations from hatchlings to the age when those vocalizations sounded mature, and songs are learned typically by juvenile males and are hormonally-dependent. The anatomical reason is that their vocalizations are produced using a specialized organ called the syrinx which does not have the multiple responsibilities of the mammalian larynx (e.g., acting as valve for respiration) [45]. Thus, there are different selection pressures for vocal production in birds versus mammals.

Adult animals must balance the limited availability of energy with their behavioral strategies. For example, in the active-sensing electric fish, swimming in a less energetically efficient manner increases the chances of encountering prey, and this increase in the prey encounter rate offsets the metabolic cost of inefficient swimming [46]. In fact, the size of the motor trajectories (i.e., the energetic cost) of at least some animals is proportional to how much information might be gained about the location of food sources by such movements; i.e., they “gamble” energy for the possibility of more accurate information [47]. During vocal exchanges, there also seems to be a similar type of foraging but in the social domain. Humans and other primates will change the amplitude of their vocalizations as a function of distance from conspecifics, getting louder when they are farther away [48,49]; louder calls require more energy [24]. Primates will also change which vocalizations they produce as a function of social distance: Marmoset monkeys will switch to shorter duration, less tonal vocalizations as they get closer to conspecifics [50,51], and this switch is directly related to decreases in energy allocation [51]. It is reminiscent of the way echolocating bats adjust the timing of their vocal output as they approach their targeted prey when foraging [52] and the manner in which male gelada vocalizations follow Menzerath’s linguistic law [53]. Our data extend the energy-information trade-off framework to the developmental time scale. During vocal development, infant vocalizations are motor trajectories and producing contact vocalizations is foraging for caregiver attention. Infant vocal sounds that are most likely to elicit vocal responses (i.e., have more information) are those that are more mature-sounding but that may be more energetically costly to produce [24,25]. Finally, it is worth noting that the energy-information trade-off we describe here may be directly related to the “compressibility and expressivity” tradeoff discussed in linguistics [54].

We only observed a single punctuation in our study. Why not more? One possibility is that other types of saltatory change occur on shorter timescales not captured at the temporal resolution of our study. For example, phase transitions can occur on the order of seconds such as those observed in human bimanual movements [55] and in gait changes in locomoting animals. In horses, at least, these transitions (e.g., from walk to trot) minimize energy consumption as locomotor speed increases [56,57]. Vocal acoustics also exhibit sudden transitions and on even shorter timescales (e.g., bifurcations and noisiness) [58,59]; these can often be related to the properties of the vocal tract (e.g., tension on the vocal organ, its material properties, and respiratory power). This may allow individuals to produce a greater diversity of vocalizations without a large increase in energy expenditure, which would be required if such vocal diversity needed exquisite neural control mechanisms [60]. On another timescale—across trials and training sessions—conditioning experiments with rodents show that learning curves are not smooth; rather they show abrupt, step-like increases in performance [61].

Beyond timescales, another possibility is that we may have masked other, more subtle nonlinear shifts in vocal development by using principal component analysis on four variables to produce a single principal acoustic parameter. Nonlinear shifts in each of those four acoustic variables may coexist at the same or different times. We know, for instance, that one contribution for the shift from noisy to tonal vocalizations in developing marmoset monkeys is the switch of sound sources from the main part of the vocal folds to the apical vocal membranes; this switch is due to the development of increased tissue stiffness [43]. Changes in vocal sound quality are also strongly influenced by respiratory power in vertebrates [13,62,63], and both vocalization duration and tonality are influenced by lung growth in marmoset monkeys [11]. In humans, lung volume almost triples over the first two years of postnatal life [64]. Finally, we may observe additional punctuations later in life, turning vocal development into a series of punctuated equilibria where different constraints are balanced. In light of this, future modeling work will have to include putative mechanisms underlying non-linear behavioral shifts during development, perhaps even taxon-specific ones (e.g., larynx versus syrinx).

Energy and information are just two variables that shape what is certainly a complicated developmental landscape. Moreover, they are sufficiently broad that they likely influence many different mechanisms—and in parallel—over the course of development. Together, energy and information may serve as a driving force, pushing, so to speak, various interacting mechanisms to progress through the necessary adaptive changes. For example, the transition from immature to mature production of contact calls in marmoset monkeys is through a combination of vocal tract growth, increased muscle strength, and increased control of related neural circuits [13]. Early in postnatal life, arousal (or the allocation of energy) is tightly linked to the production of vocalizations [28,29]. At the same time, neural circuits for vocal production are influenced by social feedback from conspecifics [10,17]. Together, arousal and social feedback influence of the development of vocal motor control [43]. Similar processes almost certainly occur in humans [65].

In sum, we show that punctuated equilibria characterize the early vocal developmental trajectories of three mammalian species. We explore and test the possibility that energy and information trade-off over the course of development to instantiate the shift between equilibrium states. Our hypotheses are supported by our manipulation experiments with marmoset monkeys. These findings highlight the important insights that focusing on the *process* of development (the “how”) can lead to, as opposed to focusing on just the outcomes (the “what” and the “when”). Focusing on the process can lead to better hypotheses about mechanisms and possible interventions when vocal development goes awry.

## Methods

All audio files used in this work were previously published. All the published experiments that generated the data used in this article were approved by the animal ethics committee from the institutes where the data were collected.

### Dataset for model fitting

We tested the model in 3 different species (common marmoset, Egyptian fruit bat, and humans) as shown in Figs 1 and 2. A total of 3 datasets were used. All datasets were composed of published data of audio files containing calls for a given species (Table A in S1 Appendix). All datasets were obtained from recordings of individual infants interacting with their caretakers or tutors. The methods are already published, but we briefly introduce them in the SI Appendix.

### Dataset for information transmission experiment

We used the interaction from infants with their parents to calculate the information transmission in Fig 4C. The interaction dataset used here is the same dataset we used for the model comparison (Fig 2) [17]. We present a summary of the data (Table B in S1 Appendix). Infant marmosets were gently separated from the adult caregiver and taken to the experiment room; the adult was brought to the room after the infant. An opaque curtain prevented the infant and the parent from having visual contact. The pair were free to vocally interact with each other [17].

### Dataset for contingency playback

We used two different contingencies to manipulate the landscapes in Fig 3F. The contingency dataset used here was reported previously [10]. We present a summary of the data (Table C in S1 Appendix). In this experiment, the parental presence was simulated by acoustic playback. Most sessions consisted of a 10 min test condition followed by a 30 min playback condition; the infants were otherwise with their families for the remaining ~23 hours each day. At each session, either the mother or father’s calls were played back (counterbalanced). One of the twins received contingent playback with low probability and the other received contingent feedback with high probability. The infants were randomly allocated to low or high contingency groups. In [10], the authors conducted experiments to compare the changes in infant vocalization due to contingency from real parental calls versus from playback calls. In both cases, the effect was the same.

### Dataset for heliox experiment

We used two different air conditions to manipulate the landscapes in Fig 4F. The heliox dataset used here was reported previously [11]. We present a summary of the data (Table D in S1 Appendix). Starting from postnatal day P1, marmoset infants were placed in an induction chamber that holds approximately 45 L of air. The subjects were introduced into the chamber through the lid on top of the chamber. Heliox (20% oxygen and 80% helium) was passed through the inlet on the chamber and air was expelled from an outlet. An airflow meter was attached to the inlet. In each session, we carried out recordings of 10 min in heliox and 10 min in air. The order of these two conditions alternated every session [35].

### Dryad DOI

https://doi.org/10.5061/dryad.f1vhhmgzv [66].

### Analysis

#### Quantification of acoustic parameters

Given the audio file of a call and before all other analyses, we calculated four acoustic properties: the call duration, the natural logarithm of the dominant frequency, the natural logarithm of the AM frequency, and the Wiener entropy. We picked these acoustic features because they are commonly extracted and analyzed in the animal vocalization literature, and they are relevant here since they change throughout development [17]. The calculation of the features used a custom-made MATLAB routine. First, the audios were filtered outside of the typical vocalization range (marmoset: 3kHz to 16kHz, bat: 2kHz to 80kHz, human: 80Hz to 1kHz). Then, the amplitude envelope was calculated with the absolute value of the spectrogram (FFT window of 1024 points) and a low pass threshold at the 90th percentile of the amplitude was applied. The call duration was calculated as the difference between the onset and the offset of the result. The dominant frequency was the median of the frequencies with the highest amplitude in each instant of a call. The amplitude modulation was filtered outside the range of dominant frequencies (marmoset: 6kHz to 10kHz, bat: 8kHz to 20kHz, human infant: 200Hz to 1000Hz), passed through a Hilbert transform and the amplitude envelope was calculated. The amplitude modulation is the dominant frequency of the amplitude envelope. Finally, the Wiener entropy (which is the broadness of the signal) was calculated by taking the difference of the mean of the logarithm of the power spectrum and the logarithm of the mean.

#### Calculation of Principal Acoustic Component (PAC)

After calculating the z-score of each property separately, we performed a Principal Component Analysis (PCA) on the acoustic properties. For the Egyptian fruit bat, we did not use the AM frequency because their vocalizations do not exhibit a significant amplitude modulation. The Principal Acoustic Component (PAC) of a call is the projection of that call (defined by their acoustic features) on the 1st component of the PCA. The 1st component and 2nd component of the PCA described 46.6% and 31.9% of the variance for the marmoset dataset, 41.0% and 27.7% for the human dataset, and 36.1% and 32.9% for the bat dataset, respectively. We restricted the analysis to only the first principal component so that it would summarize the most important dynamics of the vocal development, not considering variations that were not as characteristic of the data throughout development. Note that the PCA was applied for the whole dataset in each single species, which includes both immature and mature calls. For the marmoset data, the loadings of the PCA for the first component, after the z-score of the data, were -0.53 for the duration, 0.42 for the dominant frequency, 0.53 for the amplitude modulation, and 0.49 for the entropy. Even though there seems to be a correlation with call type, these numbers show that there is no single interpretable acoustic factor explaining the PAC. The loadings for the other animals and other components are similar (see data on DRYAD).

#### Comparison of immature and mature PAC distributions

To make sure that there is a significant difference between the vocalizations at the beginning of the recording period and the vocalizations at the end of the recording period, we compared the PAC distribution for each period. For each animal, we considered the vocalizations in the first 10% of the recording days as the immature vocalizations and the final 10% of the recording days as the mature vocalizations. We made the plot of the distributions by calculating the distribution for each individual and averaging these distributions. We used a two-sample Kolmogorov-Smirnov test using the MATLAB routine *kstest2* to verify if the PACs from the first 10% days (immature calls) would be significantly different from the PACs from the last 10% (mature calls).

### Estimation of typical PAC per day

We calculated a typical value for the Principal Acoustic Component (PAC) for the population in each day of recording (Figs 1C and 2A–2F). The typical value is defined by the peak of the distribution of PACs. Since there might be individuals with a higher amount of data, we first calculated the distribution for each individual and then we averaged it. The distributions were all calculated using the MATLAB routine ksdensity with a bandwidth equal to 0.5, regardless of the day or the species.

To avoid days with outlier behavior, we considered the other days around it instead of using a single day to calculate the distribution. That is equivalent to a data smoothing process by moving average, because one day contains information for the following (just like the following day contains information from the previous one). The size of the window of the moving average was determined by the quality of the data, and so we introduced two relevant variables: the density D that represents the number of subjects per dataset, and the heterogeneity H of the data in time, i.e., how well distributed the data collection was over time.

The variable D was calculated by:

- Taking the number of subjects and the number of calls in a single dataset;
- Dividing the number of subjects by the number of calls. Too few calls per subject should have more gaps and be more prone to outliers. Few calls per subject lead to higher variable D, increasing the window size;
- Multiplied the result by 1000 to bring the value closer to a scale of days;
- Evaluated the result by a ceil function to have an integer number.

The heterogeneity H was related to the density of recording sessions and calculated by:

- Checking the days of the sessions;
- Taking the difference between every two recording sessions, obtaining the interval between recording sessions;
- Calculating the average of these intervals. A high average interval shows data not as thoroughly sampled, which requires a bigger window size.

We used a formula for the half period size that returned values for the period that would give a reasonably smooth dataset for all animals. The half period size is the number of days before and the number of days after the day in question, i.e., the window of the moving average. The following formula was used: half-window size ⌈(D^2^*√H)/2.25⌉ where ⌈x⌉ indicates the ceil function. The values found are shown in Table E of S1 Appendix.

#### Development models

We considered two sets of calls, representing immature calls and mature calls. With these sets, we evaluated three models to study the development from immature to mature calls.

*The first model is the linear model*. In this model, the landscape shifts. Initially, the call that minimizes the landscape is the typical immature call. Then, it moves linearly so that the minimum is now at the typical mature call. Considering x as a variable representing the call acoustics (e.g. the PAC), and the developmental parameter (control parameter) as a variable λ from 0 to 1, we have that the immature landscape is C_0_(x) and the mature landscape is C_1_(x), and the landscape for a generic moment λ is C_λ_(x) = C_0_(x−λ*k) where k is calculated with the equation C_1_(x) = C_0_(x−k). The model is important to verify if the transition from the immature state to the mature state is a linear shift.

*The second model is a recurrent model*. It was inspired by Fehér et al., 2009 and considers a discrete development for λ, representing generations. In this case, the PAC distribution represented by C_λ+1_(x) in a given moment λ+1 of the development will depend on the typical PAC of the previous moment represented by C_λ_(x) weighted by a constant c_0_ and a portion of genotypic *G*_λ+1_(x) and environmental E_λ+1_(x) values. The equation representing the model is

$$C_{\lambda +1}(x)={G_{\lambda +1}(x)+c}_{0}C_{\lambda }(x)+E_{\lambda +1}(x).$$

As in Fehér et al. (2009), we consider G_λ_(x) = *N*(*G*_0_, *V*_*G*_) and E_λ_(x) = *N*(0, *V*_*E*_) as Gaussian distributions uncorrelated through generations, concluding that the expected value and variance of the distributions C are

$$E[C_{\lambda }]=G_{0}(1-{c_{0}}^{\lambda })/(1-c_{0})\;and$$

$$V[C_{\lambda }]=(V_{G}+V_{E})(1-{c_{0}}^{2\lambda })/(1-{c_{0}}^{2}).$$

To find a continuous version of the model in which we have λ ranging from 0 to 1 and the average does not start at *G*_0_, we solved the recurrence with constraints for the probability when λ = 0 and λ = 1, and obtained the following expected value

$${E[C}_{\lambda }]=k+G_{0}(1-{c_{0}}^{\lambda })/(1-c_{0}).$$

Where k is the minimum value for the PAC and gives the curve starting point, G_0_ is given by 12/(final age+⌈#subject/#data⌉) and dictates how steep the curve will raise and c_0_ is 1−G_0_/(max value for PAC−k) and gives where the curve will finish. #subject/#data is the number of subjects divided by the total number of calls in a single dataset.

*The third model is the balance model*. We consider that we have two constraints imposing different vocalization landscapes, which we will call *C*_0_(*x*) and *C*_1_(*x*). In each moment of the development, characterized by the value λ∈[0,1], we consider that the landscape C_λ_ will be given by

$$C_{\lambda }(x)=(1-\lambda )C_{0}(x)+\lambda C_{1}(x).$$

After calculating the landscape in a certain day (proportional to λ) and having a value for the temperature β, we find the probability distribution by using the softmax selection rule

$$p_{\lambda }(x)=exp(-\beta C_{\lambda }(x))/Z$$

and store the expected value of the probability distribution as the typical PAC predicted by the model. Z is the partition function, calculated through normalization. The partition function is only used to ensure *p* is indeed a probability function and should not be interpreted individually in this context.

### Estimation of the vocalization thermodynamic cost and landscape

We need to calculate the thermodynamic costs for producing a vocalization associated with certain PAC values to implement the 3 different models considered here. We can define a thermodynamic cost function c(x) that relates each vocalization with PAC = x to an associated cost to produce it [13]. Assume that this thermodynamic cost function c(x) has a fixed expected value *E*[*C*(*x*)] = *EV*. The maximum entropy principle states that we should maximize

$$H(p)=-\int_{-\infty }^{\infty }p(\theta )\;log\;p(\theta )\;d\theta \;with\;the\;constraint\;that\;\int_{-\infty }^{\infty }p(\theta )c(\theta )\;d\theta =EV.$$

According to the softmax action selection rule [13], we have that p(x) = exp(−βc(x))/Z. We can calculate the probability distribution by using the MATLAB routine ksdensity. The bandwidths used to calculate the distributions were robust—when we introduced a variation to them, the result was not altered greatly. The values used for each animal (marmoset, bat, human) for the immature distribution and the mature distribution were, respectively: 0.7 and 0.4, 0.9 and 0.3 and 0.3 and 0.6. If we already have the probability distribution, we can estimate the thermodynamic cost distribution of a call in a period given the probability distribution with *c*(*x*) = −*log*(*p*(*x*))/*β*, and the landscape was defined as the opposite of the thermodynamic cost, *C*(*x*) = −*c*(*x*). Note that this causes the landscape to be higher in regions with higher probability of vocalizations. For the energy landscape and energy thermodynamic cost, this is related to the metabolic cost of the vocalization. Importantly, what we label in this section as “information cost” is used merely to estimate the information landscape; it is not associated with the metabolic cost, despite the ambiguity of the word “cost”.

### Parameters search and model comparison

We made a search for the parameters in each model and compared the best R^2^ obtained for each to compare the goodness of fit for each model. In all models, the same three parameters were searched for the R^2^ optimization: the final immature day, the first mature day, and the “temperature” *β*. We calculated the immature PAC distribution using all the calls up to the final immature day and the mature PAC distribution using all the calls from the first mature day. Using the distributions, we calculate the landscapes. With the landscapes and the “temperature” *β*, we calculate the typical PAC through the development, including its R^2^ related to the experimental data given by the typical PAC per day.

We defined a set of possible immature and mature ranges where we can perform the search based on important developmental features for mammals. We used weaning age and sexual maturation. The values for the marmosets were extracted from Schultz-Darken, Braun, and Emborg [67]. The values for the Egyptian fruit bat were extracted from Cohen, R. (2011). The values from humans were extracted from Dettwiler and Graber [68, 69]. The values can be seen on columns 2 to 4 in Table F of S1 Appendix.

We estimated 15 days for the maximum possible age of population vocal immaturity and 40 days for the minimum possible age of population vocal maturity for marmoset monkeys. Then, we calculated a proportion of the averages (in column 5 of Table F of S1 Appendix) and estimated the maximum and minimum values for the search for humans and bats. The values are shown in Table G of S1 Appendix.

We applied a two-sample Kolmogorov-Smirnov test to determine whether this delimitation considers two different distributions for the PAC of the vocalizations emitted. For each species the p-values found were below 10^−3^, hence the immature and mature calls were significantly different for all three species.

After setting the limits of the mature and immature calls as in Table G of S1 Appendix, we made a search for the actual minimum mature day, maximum immature day, and *β* by optimizing the R^2^ separately for each animal and each model independently. The values found are shown in Table H of S1 Appendix and the best models are shown in Fig 2D–2I.

#### Transition day comparison

Once we had the best parameters for the balance model, we can compute the transition day for the model and compare it with the transition day given by the experimental data (Fig 2J–2L). We found the point with the highest slope to calculate the transition day of the model and we fit a sigmoid function using the MATLAB fit routine and the following formula for the sigmoid

$$f(x)=p_{1}+(p_{2}-p_{1})/(1+e^{(p_{3}-x)*p_{4}}).$$

to determine the transition day of the experimental data. We then retrieved the parameter p_3_ of the sigmoid function which gives the value of x where the transition occurs. We used the MATLAB *fit* routine, constraining the parameters to plausible values. The initial value for the routine was given by the values -1 and 0.5 for p_1_ and p_2_. p_3_ was initialized as the midpoint of the recording range, and the initial value for p_4_ was 1.

The same method was used to compare the transition days in Figs 3 and 4. The parameters were similar to the ones used in Fig 2J–2L. The starting point for *p*_3_ was set as 15, which was between the transitions, providing a better fitting.

To measure the confidence interval of the transition day, we resampled the data with replacement (bootstrap) and recalculated the transition days, obtaining a distribution for the transition day both experimental and predicted by the model for Fig 2 and a distribution for the transition day in each condition in Fig 4. We made a statistical test to see if we could prove that the transition days are different. The test consisted of finding the percentage of the bootstrapped transition days predicted by the model lower than the transition days predicted by the experimental data. For each bootstrap of the data, we subtracted the transition day of one context with the transition day of the other context.

#### Information transmission

We define the efficiency of information as the rate of information transmission, and it was calculated through the Granger causality. The methodology and data were reported previously [32]. We calculated the Granger causality between the onsets of syllables. Briefly, we fitted a generalized linear model to model the dynamics of the onset of syllables. We compared two models to test whether infants influence parental vocal dynamics: one in which the only predictor is the past onset times of parental vocalizations and one that considers the past onset times of both parental and infant vocalizations. If the model accounting for both parental and infant vocalizations is a better fit, we can infer that infants significantly contribute to vocal interactions with their parents. We calculated the strength of the interaction for each session and then fitted a cubic spline using MATLAB csaps for the population. We used a bootstrap method to calculate a 95% confidence interval for the population average curves. We were interested in the absolute value of the strength of the interaction, so we calculated the absolute value of those estimates.

## Supporting information

S1 Appendix Dataset for model fitting. Table A in S1 Appendix. Summary of the dataset used in the model fitting for Fig 2. Table B in S1 Appendix. Summary of information transmission experiment dataset used in the analysis for Fig 3H. Table C in S1 Appendix. Summary of contingency experiment dataset used in the analysis for Fig 4E. Table D in S1 Appendix. Summary of heliox experiment dataset used in the analysis for Fig 4J and 4P. Table E in S1 Appendix. Size of the period used to estimate the typical PAC per day for Fig 2. Table F in S1 Appendix. Values are shown in months. Developmental features found in the literature. Table G in S1 Appendix. Extreme values for possible immature and mature days range for the search in Table H. Table H in S1 Appendix. Best parameters for the linear model, recurrence model, and balance model, respectively.
(DOCX)
