# Supplementary material for: A mechanism for punctuating equilibria during mammalian vocal development
Source: PLoS Comput Biol. 2022 Jun 13;18(6):e1010173. doi: 10.1371/journal.pcbi.1010173 (PMC9232141; doi:10.1371/journal.pcbi.1010173)
Supplement: S1 Appendix — Table A in S1 Appendix. Summary of the dataset used in the model fitting for Fig 2. Table B in S1 Appendix. Summary of information transmission experiment dataset used in the analysis for Fig 3H. Table C in S1 Appendix. Summary of contingency experiment dataset used in the analysis for Fig 4E. Table D in S1 Appendix. Summary of heliox experiment dataset used in the analysis for Fig 4J and 4P. Table E in S1 Appendix. Size of the period used to estimate the typical PAC per day for Fig 2. Table F in S1 Appendix. Values are shown in months. Developmental features found in the literature. Table G in S1 Appendix. Extreme values for possible immature and mature days range for the search in Table H. Table H in S1 Appendix. Best parameters for the linear model, recurrence model, and balance model, respectively. (DOCX) [file pcbi.1010173.s001.docx]

## **S1 Appendix**

**Dataset for model fitting**. The common marmoset's infants were recorded first in an undirected context (social isolation) for ~ 5 min and then a directed context (interacting with parents) for ~ 15 min, but only the directed context was used here to maximize the influence of informational constraints. The recordings were made in a lab, with an opaque curtain to visually occlude one subject from the other. Calls were detected automatically and verified individually by humans. The Egyptian fruit bat pups were recorded during directed contexts. The pups interacted with the mother before weaning, and after weaning with other members of the colony chamber. The changes in environment are consistent with what is seen in the wild, and the transition day calculated for the experimental data of 55.88 (35.67 to 60.55, 95% CI) is consistent with previous findings [1]. The detection of the calls was made by trained experimenters. We used two different sets of data for the human vocal development to get greater coverage of the developmental periods. Both human datasets were made in the natural environments of the infants in a directed context (interacting with the mother), and in both the detection of the vocalizations were made by a researcher. The two sets overlap from PND 270 to PND 380. The transition day calculated for the experimental data was 189.25 (100.53 to 242.35, 95% CI), which is significantly lower than PND 270. Thus, we conclude that merging the two datasets was not responsible for the transition.

Note that the length of the recording sessions varies for the different protocols in the different datasets, so we define one recording session here as one day of recording, so for all species there is one recording session in the day. For humans and marmosets, the recordings were made during working hours, while for bats the recordings were taken continuously. Marmoset recordings were taken once every few days for each subject. Bats were recorded densely during a few days with longer periods without recording. Humans had sparse recording sessions throughout each infant development. For all subjects in the three datasets, their photoperiod coincided with the daylight. The gender of the subjects was not explicitly considered given the high variability of vocal behavior, which to our knowledge is not dependent on gender. That said, all of the species include both male and female individuals. The only important gender difference in the datasets to our knowledge is in bats and humans, that most of the early interactions are with female caregivers (though there were interactions with male individuals later as well).

| Species | Dataset | Number of individuals used in this article | Minimum age of recording (postnatal day) | Maximum age of recording (postnatal day) | Number of recording sessions | Total number of single calls recorded | Social context during recording |
| --- | --- | --- | --- | --- | --- | --- | --- |
| Common marmoset (*Callithrix jacchus*) | Takahashi et al., 2015 [2] | 10 (5 pairs of twins) | 1 | 60 | 60 | 105904 | Interacting with parents |
| Egyptian fruit bat  (*Rousettus aegyptiacus*) | Prat et al., 2017 [3] | 13 | 1 | 122 | 112 | 1878 | Interacting with parents and with colony |
| Human (*Homo sapiens sapiens*) | Cruz-Ferreira, [4]2003 | 3 | 30 | 380 | 21 | 476 | Interacting with mother |
| Human (*Homo sapiens sapiens*) | Brent et al., 2001 [5] | 5 | 270 | 463 | 29 | 579 | Interacting with mother |

Table A. Summary of the dataset used in the model fitting for Fig. 2.

| Species | Dataset | Number of individuals | Minimum age of recording (postnatal day) | Maximum age of recording (postnatal day) | Total number of calls recorded | Social context |
| --- | --- | --- | --- | --- | --- | --- |
| Common marmoset (*Callithrix jacchus*) | Takahashi et al., 2016 [6] | 10 infants (5 pairs of twins) and 6 adults | 1 | 63 | 117729 | Directed (Interacting with parents) |

Table B. Summary of information transmission experiment dataset used in the analysis for Fig. 3h.

| Species | Dataset | Number of individuals | Minimum age of recording (postnatal day) | Maximum age of recording (postnatal day) | Total number of calls recorded | Social context |
| --- | --- | --- | --- | --- | --- | --- |
| Common marmoset (*Callithrix jacchus*) | Takahashi et al., 2017 [7] | 6 (3 pairs of twins) | 1 | 60 | 52035 | Undirected with playback |

Table C. Summary of contingency experiment dataset used in the analysis for Fig. 4e.

| Species | Dataset | Number of individuals | Minimum age of recording (postnatal day) | Maximum age of recording (postnatal day) | Total number of calls recorded | Social context |
| --- | --- | --- | --- | --- | --- | --- |
| Common marmoset (*Callithrix jacchus*) | Zhang et al., 2018 [8] | 3 infants (one pair of twins, one singleton) | 1 | 70 | 20371 | Undirected (no social contact during the experiment) |

Table D. Summary of heliox experiment dataset used in the analysis for Fig. 4j and 4p.

| Species | Density D | Heterogeneity H | Period size (days) |
| --- | --- | --- | --- |
| Common marmoset | 1.0000 | 1.0000 | 3 (1 before and 1 after) |
| Egyptian fruit bat | 7.0000 | 1.0991 | 47 (23 before and 23 after) |
| Human | 8.0000 | 8.8367 | 171 (85 before and 85 after) |

Table E. Size of the period used to estimate the typical PAC per day for Fig. 2.

| Species | Weaning age | Female sexual maturation | Male sexual maturation | Average |
| --- | --- | --- | --- | --- |
| Common marmoset | 3 | 18 | 12 | 11 |
| Egyptian fruit bat | 2 | 15 | 15 | 10.67 |
| Human | 48 | 144 | 144 | 112 |

Table F. Values are shown in months. Developmental features found in the literature.

| Species | Maximum possible immature age | Minimum possible mature age |
| --- | --- | --- |
| Common marmoset | 15 | 40 |
| Egyptian fruit bat | 15 | 38 |
| Human | 153 | 407 |

Table G. Extreme values for possible immature and mature days range for the search in Table S8.

| Species | Maximum immature age | Minimum mature age | Betas | R² |
| --- | --- | --- | --- | --- |
| Common marmoset | 3, 13, 5 | 57, 55, 56 | 100, 100, 400 | 0.54, 0.60, 0.86 |
| Egyptian fruit bat | 36, 36, 8 | 100, 117, 99 | 500, 100, 200 | 0.81, 0.64, 0.96 |
| Human | 87, 271, 69 | 453, 337, 421 | 500, 500, 500 | 0.70, 0.75, 0.90 |

Table H. Best parameters for the linear model, recurrence model, and balance model, respectively.

**References**

1. Prat Y, Taub M, Yovel Y. Vocal learning in a social mammal: Demonstrated by isolation and playback experiments in bats. Science Advances. 2015;1(2):e1500019.

2. Takahashi DY, Fenley AR, Teramoto Y, Narayanan DZ, Borjon JI, Holmes P, et al. The developmental dynamics of marmoset monkey vocal production. Science. 2015;349(6249):734-8. doi: 10.1126/science.aab1058.

3. Prat Y, Taub M, Pratt E, Yovel Y. An annotated dataset of Egyptian fruit bat vocalizations across varying contexts and during vocal ontogeny. Scientific Data. 2017;4:170143. doi: 10.1038/sdata.2017.143.

4. Cruz-Ferreira M. Two prosodies, two languages: infant bilingual strategies in Portuguese and Swedish. Journal of Portuguese Linguistics. 2003;2(1):45-60. doi: <http://doi.org/10.5334/jpl.35>.

5. Brent MR, Siskind JM. The role of exposure to isolated words in early vocabulary development. Cognition. 2001;81(2):B33-B44. doi: 10.1016/S0010-0277(01)00122-6.

6. Takahashi DY, Fenley AR, Ghazanfar AA. Early development of turn-taking with parents shapes vocal acoustics in infant marmoset monkeys. Philosophical Transactions of the Royal Society B: Biological Sciences. 2016;371(1693):1-12. doi: 10.1098/rstb.2015.0370.

7. Takahashi DY, Liao DA, Ghazanfar AA. Vocal Learning via Social Reinforcement by Infant Marmoset Monkeys. Current Biology. 2017;27(12):1844-52.e6. doi: 10.1016/j.cub.2017.05.004.

8. Zhang YS, Ghazanfar AA. Vocal development through morphological computation. PLOS Biology. 2018;16(2):e2003933. doi: 10.1371/journal.pbio.2003933.
